# A Wireless Sensor Network for Residential Building Energy and Indoor Environmental Quality Monitoring: Design, Instrumentation, Data Analysis and Feedback

**DOI:** 10.3390/s23125580

**Published:** 2023-06-14

**Authors:** Mathieu Bourdeau, Julien Waeytens, Nedia Aouani, Philippe Basset, Elyes Nefzaoui

**Affiliations:** 1 Université Gustave Eiffel, CNRS, ESYCOM, F-77454 Marne-la-Vallée, France; 2 Université Gustave Eiffel, COSYS, F-77420 Champs-sur-Marne, France

**Keywords:** sensors, wireless, buildings, data, energy, behaviors, indoor environmental quality, retrofit action

## Abstract

This article outlines the implementation and use of a large wireless instrumentation solution to collect data over a long time period of a few years for three collective residential buildings. The sensor network consists of a variety of 179 sensors deployed in building common areas and in apartments to monitor energy consumption, indoor environmental quality, and local meteorological conditions. The collected data are used and analyzed to assess the building performance in terms of energy consumption and indoor environmental quality following major renovation operations on the buildings. Observations from the collected data show energy consumption of the renovated buildings in agreement with expected energy savings calculated by an engineering office, many different occupancy patterns mainly related to the professional situation of the households, and seasonal variation in window opening rates. The monitoring was also able to detect some deficiencies in the energy management. Indeed, the data reveal the absence of time-of-day-dependent heating load control and higher than expected indoor temperatures because of a lack of occupant awareness on energy savings, thermal comfort, and the new technologies installed during the renovation such as thermostatic valves on the heaters. Lastly, we also provide feedback on the performed sensor network from the experiment design and choice of measured quantities to data communication, through the sensors’ technological choices, implementation, calibration, and maintenance.

## 1. Introduction

Residential buildings account for a third of the overall energy consumption and greenhouse gas emissions in Europe [1]. Thus, to achieve the European objectives of energy reduction, actions such as retrofit operations must be carried out on existing buildings.

Sensors and monitoring data are a key solution [2] to diagnose buildings, verify the energy performance after refurbishment, and enrich energy building models for more representative simulations. Energy sources such as electricity, thermal energy for heating and domestic hot water, and natural gas need to be measured and monitored in buildings. This is what makes wireless sensor networks that focus on different energy end-uses and parameters important. Sensor networks have become a popular tool for in situ data collection during the past decade and have been promoted by recent regulations over energy monitoring [3] in addition to the development of new long-range and low-energy communication protocols, battery lifetime, and data processing methods.

In addition, sensor networks have been used in several domains such as user behaviors [4], testing blood sugar [5], or in robots [6]. However, this technology has been predominantly utilized in the field of energy.

However, the literature shows that the many aspects of energy-related behaviors and IEQ are difficult to target all at once and with a high level of detail [7,8], especially in residential buildings [9]. In this context, several solutions have been recently tested for onsite building operation data in both industrial and academic projects. A literature review enables the identification of two major types of instrumentation projects:

- Industrial programs such as PROFEEL (characterizing the impact of retrofit actions) or Agence Parisienne du climat (energy performance in residential condominiums). Industrial projects deploy a large number of sensors on a large number of buildings. They target the development of operative methods and broadcast the acquired knowledge for professionals from the construction and energy sectors. Because of the significant budget and human resources involved, monitoring and data analysis opportunities are superior to academic projects.
- Academic research projects. These projects focus on specific smaller-scale case studies. The academic research digs deeper into the specificity and details of a given case study and instrumentation method.

We summarize in Table 1 a sample of the reviewed projects involving the implementation of sensor networks for data collection in operating buildings.

Indoor air temperature and relative humidity are the most common measurements, supposedly because of the availability of simple components for such measurements. CO${}_{2}$ concentration and window opening detection are also popular. However, the monitoring of heating/cooling energy consumption is less frequent, specifically in academic research. In fact, data are mostly obtained from bills or large time-step manual readings. Furthermore, occupants’ behavior characterization is scarcely investigated. In the review [10], the authors have reported a diversity of occupancy monitoring techniques. However, to the best knowledge of the authors, only a few studies focused on window opening and monitoring of the occupancy. Hence, there is a significant challenge in gathering the many aspects of building energy efficiency—energy consumption, IEQ, and occupants’ behavior—in a single sensor network, which is performed in the present work.

It is also important to highlight that the number of deployed sensors is usually quite small due to limited human resources and the cost of commercial solutions. This raises two questions regarding the replicability of the work, since technologies might not be interoperable, and the conclusions of the results, because of the small number of measurements and case studies. Herein, a large number of 179 sensors were deployed in common areas and in apartments of three renovated buildings to monitor a wide variety of parameters.

The first objective of this article is to technically detail all the steps from the design, implementation of the sensors, communication protocols, data analysis, and to provide feedback for future deployments of wireless sensor networks in buildings. It can be noted that the study of building energy performance requires several months of good-quality data. However, the question of ensuring calibration over time is not raised in reviewed studies focusing on sensor network designs. Calibration is not mentioned at all in application studies while this should be an important point to address, especially for long-term monitoring campaigns, when some sensors exhibit measurement drift over time. The second objective of the article is to highlight the benefits of sensor monitoring for occupants, landlords, and engineers and researchers.

**Table 1:** Summary of reviewed studies implementing sensor networks for data collection in operating buildings.

Studies	Frei et al. [11,12]	Martín-Garín et al. [13]	Karami et al. [14]	Jankovic [15]	Guyot et al. [16]	Deb et al. [17]	Jnat et al. [18]	Jacopo et al. [19]
**Country**	Switzerland	Spain	N.C.	United Kingdom	France	Switzerland	France	Italy
**Case study**	8 single-family individual houses	1 apartment	1 computer lab	2 half-attached houses	1 mix-used building (mostly instrumented office spaces)	1 single house	3 social apartments	20 apartments
**Sensor network**	Arduino-based sensor kits	Arduino-based	Arduino-based	Wireless	BEMS system	c.f. Frei et al. [12]	Rapsberry-based	LoRaWAN-based
**Measurements**	Air temperature, relative humidity, volumetric oil flow, light pulses for electricity meters, CO${}_{2}$ concentration, luminosity, window opening	Temperature, humidity, barometric pressure, CO${}_{2}$ concentration, window opening	Dry bulb temperature, elevation temperature, relative humidity, horizontal illuminance, vertical illuminance, CO${}_{2}$ concentration, VOCs, PM2.5, occupancy	Natural gas, electricity, indoor temperature, outdoor temperature, humidity, solar irradiation, rainfall, wind speed and direction	Measurements on air handlers, radiant panels, indoor temperature, humidity, lighting, occupancy, window opening, window shading, energy system setups	Air temperature, CO${}_{2}$ concentration, inlet heating temperature, outdoor temperature, heat flow for walls and windows, window opening	Indoor air temperature, relative humidity	Indoor air temperature, pressure, relative humidity, indoor illuminance, VOC.
**Acquisition time-step**	5 min	1 min	N.C.	Daily energy consumption, 15 min for other data	5 min	5 min	30 min	1 h
**Acquisition period**	5 months	7 days	10 days	2 and a half years	N.C.	1 heating season	6 months	1 year and 1 month
**Number of sensors**	144 (18 per apartment)	N.C.	9	12	6000	16	6	40 (2 per apartment)
**Communication**	GPRS, Zigbee	WiFi	Zigbee	N.C.	N.C.	Zigbee	Radio frequencies	LoRa
**Storage**	PhP, MySQL	Google Drive excel sheets	VOLTRON sMap open-source software	N.C.	N.C.	MySQL	SD card	N.C.

In the present research article, we report a field experiment involving the deployment of a wireless sensor network for measuring energy, IEQ, and energy-related behaviors in three social housing buildings in the metropolitan area of Paris. The collected data are then used to assess the performance of retrofit actions, to obtain a better understanding of occupant comfort and usage and to propose practical recommendations in view of reducing energy consumption. First, we present the materials and methods in Section 2, where we expand the case study that contains a detailed description of the three buildings, including explanatory diagrams. We also summarize the previous works realized in this context. Moreover, we provide a technical description of the deployed wireless sensor network including the accuracy and operation range of each sensor. Then, the collected data are analyzed and discussed in Section 3. Finally, in Section 4, we provide feedback on the proposed instrumentation methodology. We enhance our technological choices and then discuss the advantages and limitations of our approach for target application.

## 2. Materials and Methods

### 2.1. Related Work

In the context of case studies of buildings equipped with sensor networks, we report the following works [20,21,22,23,24]. In [20,21], sensors were placed in an unoccupied two-story concrete building under controlled climatic scenarios using the Sense-City climatic chamber [25]. In [22,23,24], the authors describe a typical method for implementing variable ad hoc networks. We also report in Table 1 detailed studies in operating buildings from different countries and based on different types of sensor networks with variable acquisition time-steps.

### 2.2. Case Study

#### 2.2.1. General Description

**Figure 1:**
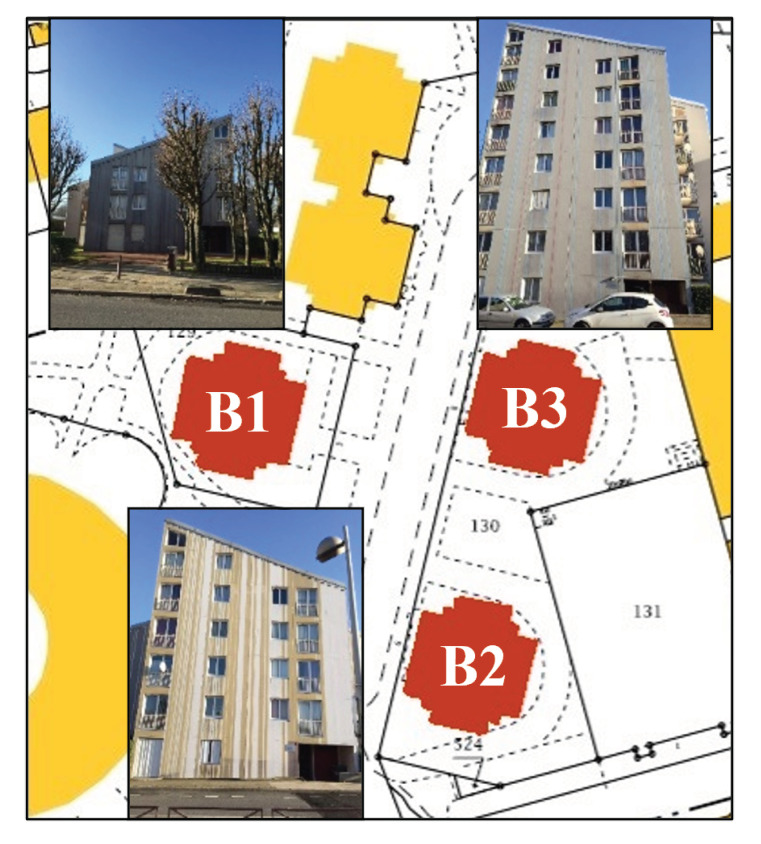
Neighborhood plan and pictures of the facades of buildings before the retrofit in Seine-et-Marne, France.

The case study is a group of three existing and occupied residential buildings located in Seine-et-Marne, in the greater Paris area. The three buildings built in 1974 have sixty-three social housings, for a total living area of 3825 m${}^{2}$. In the following, buildings are referred to as B1, B2, and B3 (Figure 1). They have thirteen, twenty-one, and twenty-nine housings, and living areas of 765 m${}^{2}$, 1275 m${}^{2}$, and 1785 m${}^{2}$, respectively. A description of the housing characteristics is given in Table 2.

The instrumentation campaign at the building scale is deployed on energy systems, in shared areas, and in an eight-apartment sample. The distribution of instrumented housings is presented in Table 3.

#### 2.2.2. Summary of the Buildings’ Characteristics and Retrofit Actions

All three studied buildings underwent an extensive energy retrofit from July 2020 to July 2021. The landlord, *Marne-et-Chantereine Habitat*, also built at the same time a new building located nearby and where the current boiler is now located.

The building envelope was fully insulated, including outdoor walls and ceilings and walls that separate heated areas from nonheated areas. The roof was not modified since its existing thermal features were sufficient for the current energy efficiency requirements. The ground floor, above the crawl space, was not insulated since it could not be properly accessed during the retrofit. All windows, glazed doors, outdoor doors, and apartment entrance doors were changed. An entrance air lock was also created at the main entrance of the buildings.

Heating and DHW production systems were already central. Prior to retrofit actions, heating and domestic hot water were produced by a neighborhood furnace using natural gas. The furnace served the three buildings as well as dozens of other social collective housing buildings owned by another landlord. During the retrofit, *Marne-et-Chantereine Habitat* opted for a more cost-effective solution. A furnace was built in their new building and connected to the extended geothermal heating network of the city. Ventilation was replaced by a new humidity-sensitive simple-flow CMV (Controlled Mechanical Ventilation) in each building and new extraction units were installed in apartments.

Common areas of the building do not have any heaters or ventilation. Appliances include lifts (only for B2 and B3) and occupancy-driven lighting with a timer. In housing, heaters were replaced with new models including a thermostatic valve, and the main hot water pipes were insulated, as well as part of the secondary piping circuit. Electric systems and switchboards were rehabilitated.

#### 2.2.3. Description of Housings

There are four types of apartments in the three studied buildings, with a main bedroom/living room or one, two, and three bedrooms. The architectural features of the housings are similar within and between buildings. There are four apartments per floor, except for the ground floor, with three apartments, and the last floor, with two apartments. Each housing is located in a corner of a floor and has a single orientation: northwest, northeast, southeast, or southwest.

Apartment energy meters (resp. water meters) include electricity and natural gas (resp. tap and hot water). Natural gas is only used for cooking but some apartments have electrical cooking appliances and do not use natural gas. Heaters are located in each room, except in the bathroom and water closet; apartments on the last floor have a heater in the bathroom. Controlled Mechanical Ventilation (CMV) extraction units are located in the bathroom, the water closet, and the kitchen.

The instrumented eight-housing sample was selected to account for a diversity of sizes, locations in the buildings, orientations, and residents’ profiles and is summarized in Table 3. In the following, the housings are designed by their building and floor, e.g., B1/3 corresponds to the apartment in building B1 on the 3rd floor. The socioeconomic information given in Table 4 also reveals the variety of occupants’ profiles in instrumented housings.

### 2.3. The Wireless Sensor Network

#### 2.3.1. Target and Deployment

Wireless sensor networks, deployed for research purposes, focus on different energy end-uses and parameters, both at the building and household levels. Measurements are divided into four categories: (i) the energy consumption with thermal energy, electricity, and natural gas, (ii) the IEQ (Indoor Environment Quality), (iii) the occupants’ behaviors, and (iv) the local weather. Energy consumption monitoring is one of the main goals of this study. Energy sources include electricity, thermal energy for heating and domestic hot water, and natural gas for cooking. IEQ focuses on parameters that depict the occupants’ comfort [26] and the thermal characteristics of the building. Both are related to building energy consumption [27,28]. Occupants’ behavior is also a significant energy driver [29]. Finally, the local weather impacts building energy needs, specifically its thermal energy consumption. Because of the diversity and the number of sensors, the deployment of the instrumentation solution is divided into two steps. The final schedule is the following:

- Step 1: instrumentation in common areas and building-scale measurements, including the IEQ, the occupants’ behavior in common areas, the building-scale electricity and thermal energy consumption monitoring, and the weather station;
- Step 2: instrumentation in households, including the IEQ, occupants’ behavior and energy consumption (thermal, electricity, and natural gas).

The full solution deployment took over two and a half years from January 2019 to July 2021. Sensors and communication protocols from the first step were also used as a test phase to highlight practical issues and conclude on the use of sensors for specific data acquisition. We should note that the proposed network does not have automatic error correction or repair capabilities. In the following sections, preparation of the sensor network is briefly summarized, and the sensor network is detailed for each type of measurement. Common areas and housings are discussed separately. Data communication and storage are described in a separate section.

#### 2.3.2. The Measurement Campaign Preparation

A design brief was created to specify the needs and purpose of the instrumentation. It is a reference document for all actors of the sensor network deployment, either from the research team or contractors providing equipment and sensors. It evolved through discussions with the contractors to fit the reality of the IoT market regarding existing instrumentation solutions and costs.

Once the design brief was ready, three actions were led simultaneously. First, households willing to participate in the experiment were recruited. This was one of the major challenges of the research project since the participation of residents was voluntary. In exchange for their participation, collected data are returned to residents with a dedicated overview of their apartment and energy consumption. Only nine housings agreed to participate, which was later reduced to eight housings since one family moved out of their apartment during the measurement campaign and was not replaced.

The recruitment campaign has highlighted that most residents did not give significant attention or make substantial efforts toward energy efficiency, either because they were not aware of the topic or because they were mostly focused on the retrofit of their apartment in terms of comfort. A few households were also concerned with the use and security of their data and electrosensitivity issues.

Then, the objective was to select one or several contractors to provide sensor network solutions. The strategy was to keep the number of involved contractors to a minimum, to minimize potential project management issues. After a large survey, thirteen contractors provided a commercial offer. Four contractors were immediately disqualified due to budget constraints. Seven contractors moved into a second round to refine their offer based on the detailed needs of the project. Two contractors were finally selected to provide the equipment, instrumentation installation, partial management of the sensor network and the weather station. Part of the setup, the installation, and the supervision were performed by our research team. Finally, we focused on the communication with the French CNIL organization (Commission Nationale Informatique et Libertés). It was required to provide all details of the project related to data collection, analyses, and future use, to protect the residents’ privacy. CNIL advised on potential warnings and provided recommendations. For similar projects, if a declaration on data usage is mandatory, the implementation of recommendations is not. For the present project, most comments concerned the following:

- The relationship between the landlord and other project actors;
- The communication means and documents regarding the research project and the residents;
- Collected data usage: potential use outside of the European Union (resulting in modified regulations), storage specifications, data transmission means, and residents’ personal data transferred to the landlord—both the researchers and the landlord are responsible for collected data use, since the data are related to residents living in the landlord’s buildings, despite data being processed by the research team only;
- A written residents’ agreement.

#### 2.3.3. Data Communication and Storage

The sensor network is entirely wireless and it relies on two different communication protocols. The first protocol is GPRS (General Packet Radio Service), a standard data package communication protocol. The second protocol is LoRaWAN (Long Range Wide Area Network). It is a radio communication protocol commonly used for smart city applications [30] and relies on LoRa peer-to-peer technology to connect communicating objects. It offers advantages over other competing communication protocols with long-range communication and very low energy consumption. Within the sensor network, LoRaWAN is divided between an operated network and two private networks.

Operated LoRaWAN networks are managed by national telecommunication companies. This option has a major benefit with a simple and straightforward implementation. Communicating devices are declared online and a fee is applied for each communicated data point. Data preprocessing such as identifying missing data, treating duplicates, and formatting, is performed on the operator’s servers. However, operated LoRaWAN networks are constrained regarding bandwidth usage, which limits potential applications (such as small acquisition time-steps or large numbers of sensors). For the implementation of an operated LoRaWAN network, gateways are used as data communication relays to the operators’ servers.

Private LoRaWAN networks rely on the same technology but the network is a local network. LoRaWAN communication is implemented between sensors and a dedicated gateway. The gateway decodes radio packages and transfers the data to a private server using the internet. Hence, there is no constraint regarding data acquisition. However, each sensor is connected to a specific gateway with a higher risk of data loss in case of a malfunction, and the initial investment cost is more significant than with operated networks, specifically because of the cost of the gateways.

**Figure 2:**
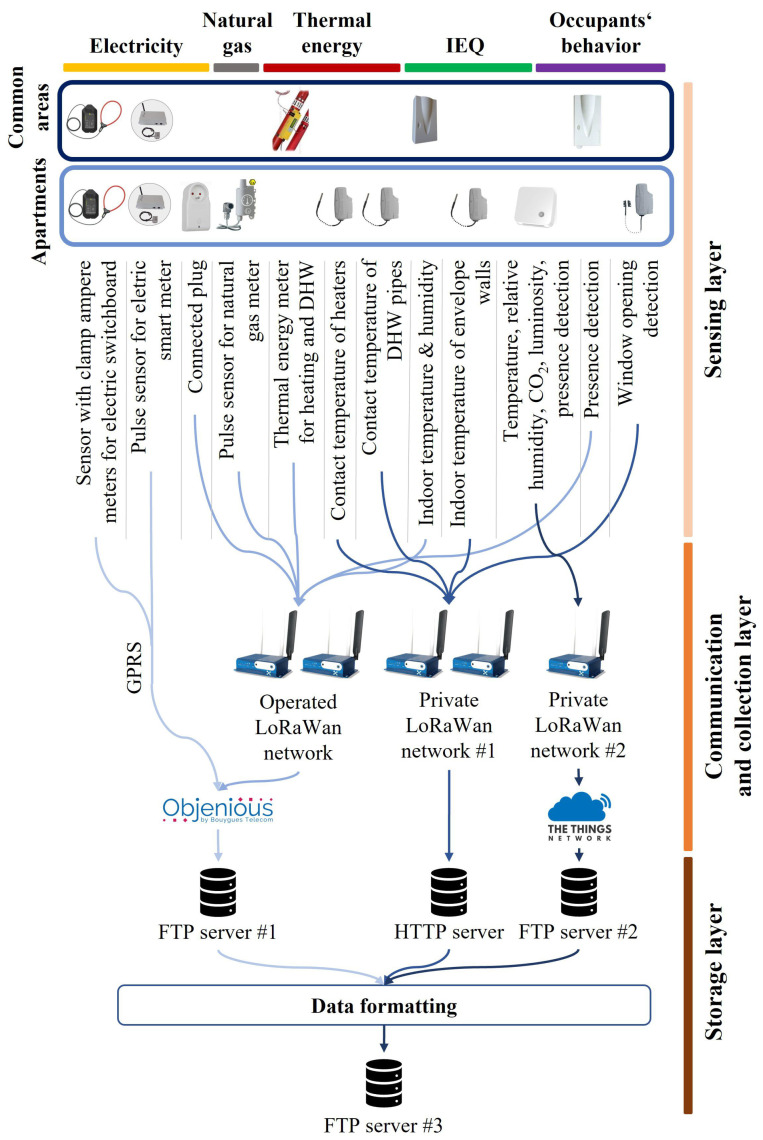
Description of the sensor network from sensors to data storage with three layers. The sensing layer includes all meters and sensors, the communication and collection layer relates to gateways and data processing platforms from Objenious [31] and The Things Network [32], and the storage layer groups all ftp and http storage servers.

The main part of the sensor network relies on the LoRaWAN protocol as illustrated in Figure 2. The LoRaWAN operated network depends on two gateways for sensors installed in common areas, along with pulse sensors for gas meters and connected plugs in housings. Two different private LoRaWAN networks are implemented. The first (private LoRaWAN network #1 in Figure 2) is dedicated to sensors for IEQ monitoring (temperature, humidity, luminosity, and CO${}_{2}$) and presence detection in apartments. The second private network (private LoRaWAN network #2) connects all remaining sensors located in apartments. It depends on two gateways due to the number of connected sensors. GPRS data communication is used for electrical measurements on electric smart meters and switchboards. All gateways are installed in technical rooms on the ground floor of the buildings. The network does not have automatic error correction or repair capabilities.

Prior to data storage, part of the collected data is preprocessed through network servers. The GPRS and operated LoRaWAN network are processed through Objenious [31], the national LoRa company from Bouygues Telecom. IEQ data are managed through the platform of The Things Network [32]. Data from the private LoRaWAN network #1 are processed online by our research team.

Collected data are initially stored on separate servers due to sensor technologies and deployment management during the research project. The largest part of the data is stored on FTP (file transfer protocol) servers except for data from the LoRaWAN private network supporting the window opening detection sensors. Window-related data collection is event-driven. Therefore, an HTTP (hypertext transfer protocol) server is needed. Data processing is performed to store all collected data on a single FTP server (FTP server #3 in Figure 2) in CSV (comma-separated values) files.

#### 2.3.4. Measurements

**Figure 3:**
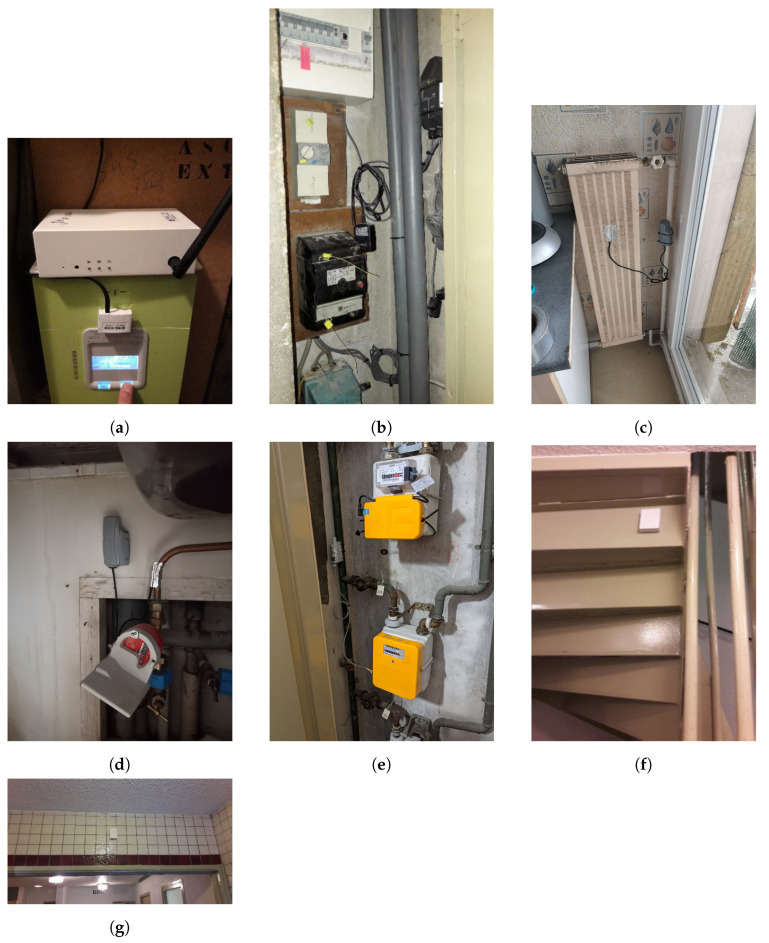
Installed sensors. (**a**) Pulse sensor installed on a Linky smart meter. (**b**) Sensor with clamp ammeters for submetering on an electrical switchboard. (**c**) Temperature sensor for heater surface temperature in apartments. (**d**) Temperature sensor for DHW pipe temperature. (**e**) Pulse sensor for a natural gas Gazpar smart meter. (**f**) Temperature and humidity sensors installed in shared building areas. (**g**) Presence detection sensor.

Measurements are divided into four categories: local weather, energy consumption, IEQ, and occupant behavior. The following sections detail the instrumentation for each type of measurement. The different types of sensors, their number and their characteristics, including accuracy and operating range, are summarized in Table 5, Table 6 and Table 7. Photos of the different sensor installations are summarized in Figure 3.

**Figure 4:**
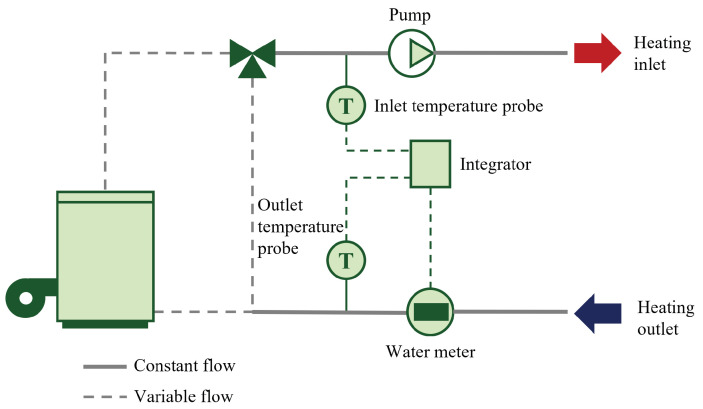
Schematic of a thermal energy meter for heating or DHW energy metering. Adapted from (Appendix A).

1. Local weather.
  - Context: Reliable quality weather data are needed for a consistent energy analysis. A dedicated weather station was acquired and deployed for this purpose. With a reasonable budget, it provides customized data collection with little day-to-day supervision and an autonomous power supply.
  - Technical specifications: The weather station monitors outdoor air temperature, relative humidity, rainfall, wind speed, wind direction, and atmospheric pressure. Two sensors are added: a black globe to measure the radiant temperature, and a pyranometer to measure the incident solar radiation intensity. Specifications of the sensors are summarized in Table 6.
  - Setup: The weather station is set up on the roof of a university building, three kilometers away from the instrumentation site. Installing the weather station directly onsite would have been the optimal solution. However, the local building configuration could not enable simple and secure access to a roof for occasional maintenance. The weather station data collection and transmission are entirely managed by a data logger using GPRS data communication. The data acquisition and communication time-steps are set to five minutes. Data are stored on a dedicated FTP server and on the cloud storage of the manufacturer. The latter solution provides an online visualization platform of the collected data. The weather station has an autonomous power supply, including a photovoltaic solar panel and a battery.
2. Energy consumption: electricity.
  - Common areas:
    Electric appliances at the building scale include elevators, lighting, CMV, and hot water pumps. Linky smart electricity meters are already installed onsite. Although Linky data can be collected through a specific process with the utility company Enedis (see Appendix A), data granularity is thirty minutes at the lowest. A smaller acquisition time-step is expected to capture small triggering events. Hence, Linky meters are instrumented using a pulse sensor (Figure 3a) to count the number of light pulses; one pulse equals one unit of electricity consumption (in Wh). Data are acquired and transferred at a one-minute time-step using the GPRS communication protocol.
    Sensors are installed in technical rooms. In all three buildings, there is one sensor for the elevator, CMV, floor lighting, elevator lighting, and the magnetic entrance door. A meter is also used for the hot water pumps of the heating substation and later removed when the heating substation is modified following energy retrofit actions. Pulse sensors are powered with batteries.
    The main electrical switchboard of B3 is instrumented with a sensor connected to six clamp ampere meters (Appendix A). It is shown in Figure 3b. It measures the current of indoor lighting in shared areas and calculates the corresponding energy consumption, assuming a constant 240 V voltage and single-phased current (C = 0.9). Data are collected and transferred with a ten-minute time-step using GPRS. A dedicated power supply and circuit breaker are required for this sensor.
  - Housings: In housings, electricity monitoring targets two spatial scales for data collection. Pulse-reading sensors for Linky smart meters are installed in each instrumented apartment. They monitor the overall electricity consumption of the apartment. Sensors with clamp ammeters for electrical switchboards are also used. Only three of these sensors are installed, in B1/3, B2/0, and B3/0, because of the intrusive data collection process, the difficult installation, and the cost of the sensors and their installation. The supervision of the electricity switchboard focuses on room-aggregated electricity consumption, lighting, and large appliances whose electricity consumption cannot be accessed by other means. The electricity monitoring system is complemented with connected plugs (Appendix A) that measure the energy consumption of household appliances. They are used to represent the energy consumption of the apartments at the appliance level. The number of smart plugs depends on the configuration of the households and the identified appliances.
    Data collection is performed using the operated LoRaWAN network with a one-minute acquisition time-step and a ten-minute data communication time-step. Hence, electricity consumption can be analyzed at three different scales: apartment, room, and appliance.
3. Energy consumption: thermal energy.
  - Common areas: Thermal energy consumption refers to hot water production for heating and domestic use. It is monitored using specifically designed thermal energy meters. Thermal energy meters have three distinctive elements (Figure 4): (i) two temperature probes, one on the inlet and one on the outlet water pipe, (ii) a flow meter installed on the outlet water pipe, and (iii) a computer to calculate the corresponding thermal energy consumption using temperature and flow measurements with respect to features of the piping system.
    Implemented thermal meters use ultrasonic water flow probes that do not require an intrusive integration into the water circuit. They are simply installed on the water pipes. Ultrasonic meters measure the time needed by an ultrasonic impulsion to move from the sending probe to the receiving probe through the water pipes. Knowing the traveling time and depending on the inner diameter, outer diameter, and material of the pipe, the water flow is assessed. Temperature probes are located on the outside of the pipe and are insulated from the surrounding environment.
    Thermal energy meters available on the IoT market are not communicating meters, and it is necessary to add a pulse sensor (Appendix A). As for electricity meters, this sensor counts the number of pulses from the energy meter (one pulse equals one kWh of thermal energy consumption). Thermal energy meters require a power supply with a circuit breaker, and the pulse sensors are powered with batteries.
    Thermal energy consumption data are collected for relatively large-time-range analyses (daily, monthly, or annual). Hence, an hourly acquisition and transmission time-step is established for all thermal energy meters using the operated LoRaWAN network.
  - Housings: Thermal energy in apartments is characterized considering heaters and DHW pipes, but it is more difficult to characterize for building-scale data acquisition. Ideally, measurements for both end-uses should be performed on the main hot water pipes with thermal energy meters. However, these pipes can hardly be accessed and such an instrumentation setup would be too expensive for consideration.
    Contact temperature sensors (Appendix A) are installed on all heaters of each instrumented apartment. See Figure 3c. Measurements are performed on a private LoRaWAN network with thirty-minute data acquisition and communication time-steps.
    For DHW, the same sensor given in Figure 3d is used with a one-minute acquisition time-step and a twenty-minute communication time-step. The small data acquisition time-step aims to precisely capture DHW consumption through the variations in DHW temperature. The sensor is installed on the outlet of hot water meters in the apartments.
4. Energy consumption: natural gas. Natural gas is used only in apartments for cooking. Natural gas meters are equipped with Gazpar modules (see documentation in Appendix A). As for the Linky smart electric meter, the Gazpar meter is a smart meter implemented by the national utility GRDF (*Gaz Reseau Distribution France*) and collects consumption data. A pulse sensor is used on Gazpar meters (Figure 3e). It reads every pulse corresponding to a 10 dm${}^{3}$ change in gas consumption. Unlike other pulse sensors, it is specifically adapted to be used in explosive atmospheres. A total of four sensors are implemented with a one-hour data acquisition and communication time-step on the operated LoRaWAN network.
5. Indoor environment quality (IEQ).
  - Common areas: In common areas of the buildings, IEQ is characterized through Indoor Air Temperature (IAT) and relative humidity measurements. For each building, three sensors are installed: one on the ground floor, one on the intermediate floor and one on the last floor. Preferably, these sensors would be installed at a height of 1.50 m. However, since sensors are installed in corridors, they need to be out of sight and reach. Sensors are located under the ceiling, on the structure of the stairs (Figure 3f).
    The data acquisition time-step is set to one hour. No sudden variations in temperature and humidity are expected, apart from doors or windows that could be opened near the sensors. Data are communicated with an hourly time-step using the LoRaWAN operated network.
  - Housings: IEQ measurements in housings are IAT, relative humidity, brightness, CO${}_{2}$ concentration (Appendix A), and occupants’ presence detection, which is described below. The sensors are located in the living room of housings. Measurements are performed with a half-hour time-step on a private LoRaWAN network (private LoRaWAN network #2 in Figure 2).
    Another sensor measures the contact temperature of the inner surface of walls. The sensor is the same as for heaters and DHW. These data aim to assess the radiant temperature of walls, which is a relevant parameter regarding indoor comfort; even though the air temperature might be sufficiently high, if the surrounding walls are excessively cold, it affects the feeling of comfort [33]. These data can be combined with IAT measurements to calculate the operative temperature, the average between IAT and the indoor temperature of walls. These sensors are set up with hourly data acquisition and communication time-steps on the operated LoRaWAN network.
6. Occupants’ behavior.
  - Common areas: Occupants’ behavior characterization in building common areas is performed with infrared presence detectors (Appendix A) positioned above the main entrance door of the buildings (Figure 3g). This technology is commonly found in automated lighting systems linked with timers [10]. PIR sensors do not discriminate between people entering or leaving the building and they cannot count the number of detected people. Presence detection is aggregated and communicated at an hourly time-step using the operated LoRaWAN network.
  - Housings: Occupants’ behavior in households is characterized through presence detection in the apartments (from the same multimeasurement sensor used for IEQ monitoring (Appendix A)) and window and glazed door opening detection. Presence is assessed using an infrared sensor as in common portions of the buildings. Data are aggregated at a half-hour time-step. It counts the number of passes in front of the sensor.
    Window opening detection is monitored with contact sensors (Appendix A). Data acquisition and communication are event-driven; data are acquired and transferred when the window opening status changes. Then, if there is no status change for over an hour, a data point is sent every hour to recall the latest opening/closing status of the window.

More details on the exact location of the sensors inside a specific apartment can be found in [34].

## 3. Data Analysis and Key Learnings

In this section, the collected data from the wireless sensor network are analyzed to verify the expected building energy performance after renovation and indoor environmental quality and to obtain a better understanding of occupancy and usage.

### 3.1. Heating Energy Consumption of the Buildings

**Figure 5:**
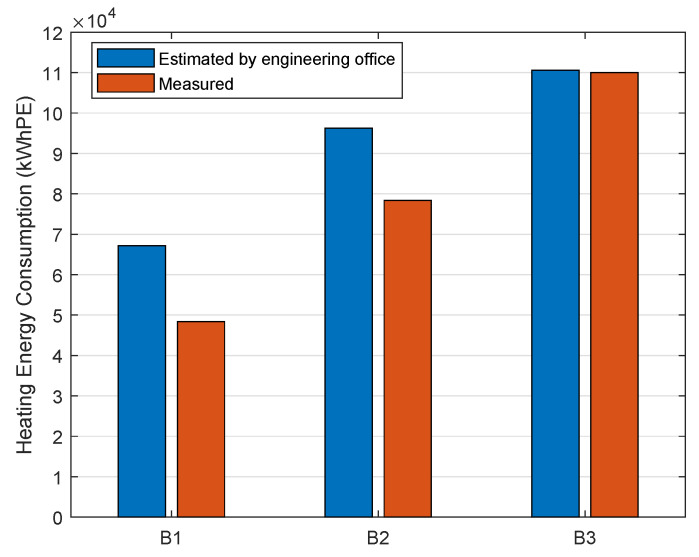
Annual heating energy consumption of the three renovated buildings from engineering office estimation and from measured data in 2021 using the wireless sensor network.

Before the retrofit actions, energy performance diagnosis (EPD) was performed for the three buildings by an engineering office. The bad grade of “D” was given as their total estimated primary energy demand ranged from $274\;{\mathrm{kWh}/m}^{2}/\mathrm{year}$ for Building 2 to $285\;{\mathrm{kWh}/m}^{2}/\mathrm{year}$ for Building 3. By the retrofit operations, the goal was to reach the “B” grade level of the EPD. The EPD on renovated buildings gives a total estimated primary energy consumption of $88\;{\mathrm{kWh}/m}^{2}/\mathrm{year}$ for Building 1, $84\;{\mathrm{kWh}/m}^{2}/\mathrm{year}$ for Building 2, and $83\;{\mathrm{kWh}/m}^{2}/\mathrm{year}$ for Building 3, which corresponds to 101,834kWh/year, 151,731kWh/year, and 187,165kWh/year for the overall estimated primary energy consumption of Buildings 1, 2, and 3, respectively. Regarding the different energy contributions estimated by the engineering office, approximately 63%, 27%, 7%, and 3% are associated with heating, DHW, lighting, and auxiliary, respectively. Thanks to the monitoring system, the heating energy consumption for each building was measured and then compared to the estimated values given by the engineering office in Figure 5. We observe that the measured heating energy consumption in 2021 in B1, B2, and B3 is 1% to 28% lower than the estimated heating values.

In conclusion, the deployed wireless sensor network allows us to verify that the performance target for heating consumption was achieved.

### 3.2. Indoor Air Temperature in Apartments and Heating Patterns

**Figure 6:**
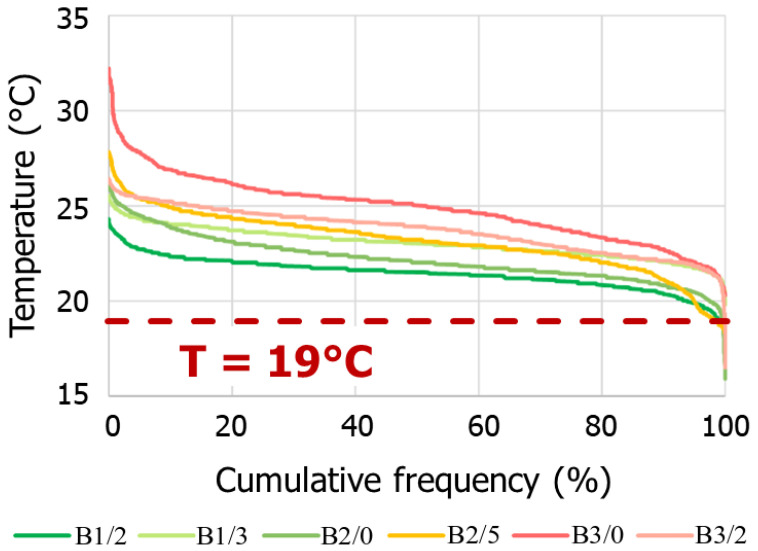
Cumulative frequency curves of measured air temperatures in the living room of instrumented apartments.

Heating patterns are characterized using indoor air temperature and heater temperature measurements. Indoor temperature measurements show that the obtained measurements are much higher than the recommended 19 °C setpoint temperature. Indeed, the average indoor temperatures in the apartment living room range from 21.4 °C for B1/2 to 24.9 °C for B3/0 for the 2021–2022 heating season. As shown in Figure 6, approximately 98% of the temperature measurements are above the 19 °C threshold.

**Figure 7:**
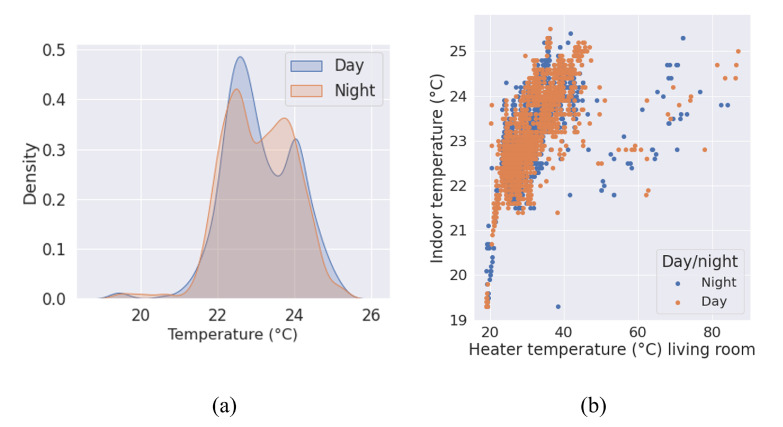
Density curve for indoor temperature in B1/3 (**a**) and scatter plot of indoor temperature vs. heater temperature in the living room of B1/3 (**b**) comparing daytime and nighttime measurements.

Despite the fact that the three residential buildings are previously retrofitted with a brand new heating system, the analysis results show that there is no water logic management of the heating system. Indeed, there is no difference between day and night temperatures in the apartment (Figure 7a), which is also confirmed by the heater temperature analysis (Figure 7b).

During the renovation operations, thermostatic valves that can be manually controlled by the occupants were installed on the heaters of the apartments. Nevertheless, the residents were not aware of the good use of this new system and they had no indication of their housing indoor air temperature. Consequently, discussions with occupants revealed that they never modify the thermostatic valve position except when thermal discomfort due to excessive temperature was felt, especially in the bedroom. In conclusion, the deployed monitoring system identified weaknesses in the building energy management, i.e., higher than the standard air temperature in apartments and the absence of heat regulation at night. Basic indoor temperatures should be provided in the apartments and the landlord should organize information campaigns to raise the occupants’ awareness of energy savings and their thermal comfort and to help them in the use of the newly deployed thermostatic valves.

### 3.3. Occupancy and Dissipated Electric Power Consumption in Apartments

**Figure 8:**
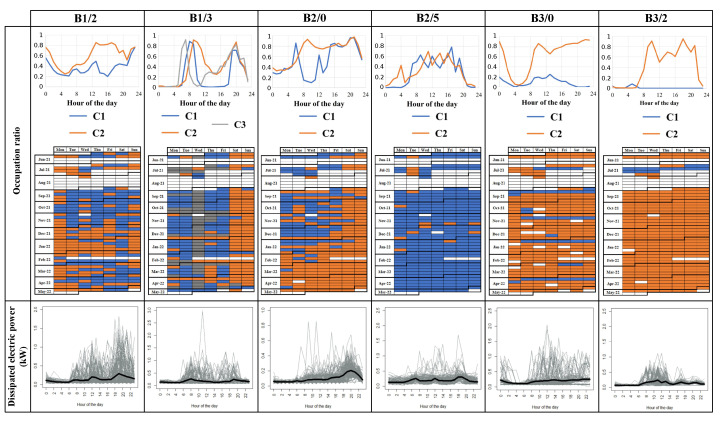
Clustering of occupancy in the living room and of the dissipated electric power in the apartments. C1, C2, C3 denote the different determined clusters.

Building occupancy can be a predominant energy driver leading to DHW consumption, window opening, dissipated power from appliance use, etc. Occupancy is deduced from presence detectors in the living room of the apartments. Clustering is performed to extract the main occupancy profiles for each instrumented apartment. The clustering process is adapted from previous works of the authors on daily load profile characterization [35]. The mean occupancy profile for each cluster (noted C) in the different apartments and their associated period are given in Figure 8. Three typical daily profile distributions can be distinguished: (i) workdays (C1) versus weekends/days off (C2) observed in apartments B1/2 and B2/0, (ii) workdays (C1), half workdays (C3), and weekends (C2) in apartment B1/3, and (iii) full presence over the day in apartments (C1 for B2/5 and C2 for B3/0 and B3/2) and potential period of absence (C1 for B3/0 and B3/2) in apartments B2/5, B3/0, and B3/2. The situation of the occupants given in Table 4 corroborates the clustering results. The occupancy profiles (i) and (ii) are associated with workers, whereas (iii) corresponds to retired (in B3/0 and in B3/2) or unemployed persons (in B2/5). Moreover, the clustering was also able to determine a change in occupancy behavior that occurred in apartment B2/0 in January 2022. In fact, the questionnaire and the survey of the building residents revealed that the person in B2/0 had sick leave in January 2022.

Concerning the electric dissipated power, it is deducted from electric power demand at the apartment scale, measured on smart electricity meters. Figure 8 shows the clustering conducted on daily dissipated electric power from May 2021 to November 2021. For all the studied apartments, only one dominant cluster is identified. Contrary to the daily occupancy profiles, characteristic and sharp dissipated electric consumption patterns are not found. Only small peaks can be observed at breakfast, lunch, and dinner times, which is in agreement with the occupation ratio profiles. This may be because the inhabitants vary their use of electric devices from one day to the other. Nevertheless, two lessons can be drawn from the measurements. First, the dissipated electric power amplitudes give an overall indication for each housing on the level of household appliances and their intensity of usage. The occupant survey underlined a high heterogeneity between the apartments with regard to the possession of electric devices. The survey confirmed that much household equipment is present and used by the family in B1/2, by the worker in B1/3, and by the couple of retired persons in B3/0. The old retired person in B3/2 had low electric usage despite the large amount of equipment she owned; on the other hand, the private sector employee with six pets in B2/0 and the unemployed person in B2/5 have few electric devices and low usage. Moreover, the occupants mentioned that they stopped using auxiliary heaters after the retrofit operations, which allowed them to reduce dissipated electric power consumption. Second, the dissipated electric power measurements indicate that almost all apartments do not take advantage of attractive pricing of off-peak-hour electricity between 11 p.m. and 7 a.m.

To conclude, using the monitoring data in studied buildings and the clustering, we gain a better understanding of occupancy patterns and overall electric equipment usages, which can be valuable for building energy modeling in view of having more realistic thermal dynamics simulations and energy predictions.

### 3.4. Natural Ventilation and CO${}_{2}$ Measurement in Apartments

**Figure 9:**
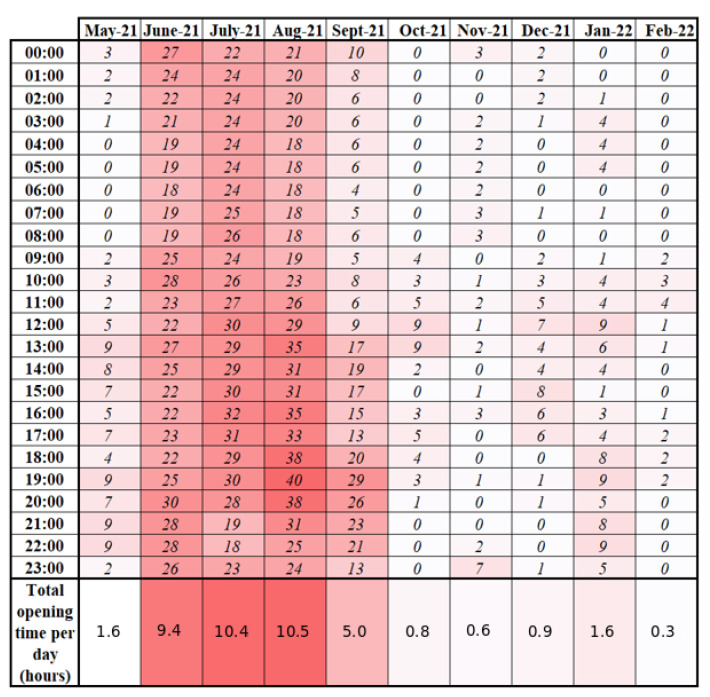
Daily window opening profiles for each month from May 2021 to February 2022. Each cell shows the number of minutes of opening for each hourly time slot in the living room for the B1/2 apartment.

Natural ventilation is pictured by window opening. Window opening data are processed to produce daily profiles with an opening duration for each hourly time slot of the day. Daily profiles are then aggregated for each month. The data show that this behavior is mostly season-driven as for the example of the living room in B1/2 (Figure 9). Windows are mostly opened during the summer period. This can be explained by the absence of air conditioning units in the buildings. Furthermore, regarding the short time of opening during the heating season starting in October, low window opening duration may not have a very significant impact on heating energy consumption.

**Figure 10:**
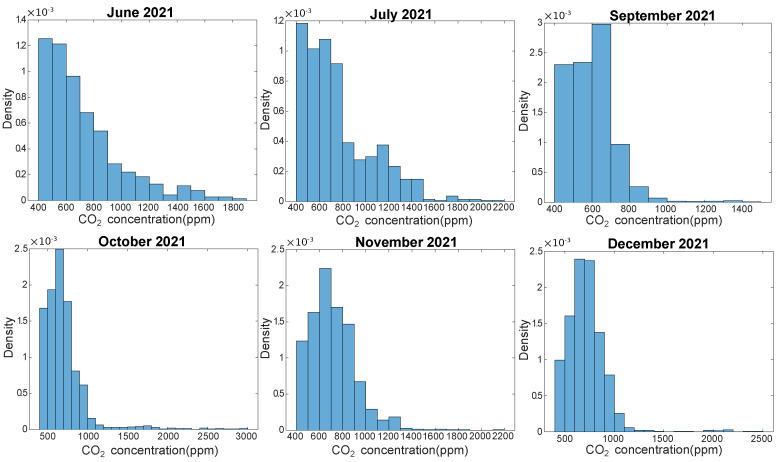
Distribution of the CO${}_{2}$ concentration for each month between June 2021 and December 2021 in the living room of apartment B1/2. August 2021 is not represented due to data loss.

To study the impact on air quality of a low window opening duration, which is encountered in fall and winter seasons, we analyze CO${}_{2}$ measurement data collected in the living room of the B1/2 apartment from June 2021 to December 2021. Indeed, the indoor CO${}_{2}$ concentration is a good indicator of the level of air confinement, which depends on human occupancy and air ventilation. Figure 10 represents the distribution of CO${}_{2}$ concentration in the living room for B1/2 for each month. We observe two types of distributions. On the one hand, in June and July 2021, when window openings are significant, the largest amount of CO${}_{2}$ data is in the category [400 ppm, 500 ppm], which is close to the outdoor CO${}_{2}$ concentration and the distributions decrease with increasing indoor CO${}_{2}$ concentrations. On the other hand, in fall and winter months (September to December 2021) with rare window openings, the indoor CO${}_{2}$ distributions have a bell shape where the category [600 ppm, 700 ppm] has the highest amount of data. The month of September can be seen as a transition of seasons with a behavior mixing the two precedent distributions. Hence, the seasonal modification of window openings and of natural ventilation can be observed in the CO${}_{2}$ concentration measurement. Nevertheless, it is important to highlight that despite the almost nonexistent window opening in fall and in winter, the indoor CO${}_{2}$ concentration in the living room in B1/2 remains at a good level. Indeed, whichever the month, the CO${}_{2}$ concentration is lower than 1000 ppm more than 80% of the time, whereas the CO${}_{2}$ concentration exceeds 1700 ppm less than 1% of the time. To conclude, sensor monitoring allows us to verify through CO${}_{2}$ measurements that the new ventilation system installed in the apartment during the retrofit operations performs correctly and ensures proper air renewal in the apartment.

## 4. Feedback on the Implementation and Operation of the Sensor Network and Possibilities for Improvement

Feedback on the deployed sensor network is presented from the experimental design and choice of measured quantities to data communication, through sensor technological choices, implementation, calibration, and maintenance.

### 4.1. Feedback on the Deployed Sensor Network

The deployment of our sensor network provides valuable feedback on the main achievements and difficulties in the implementation of an instrumentation solution. Critical points to take into account can be summarized in three categories and are further described in [36]:

- *Installation conditions and environment.* The case study is a group of three existing buildings built in 1974. The integration of a sensor network in existing buildings is more difficult than that in a newly built or recently built structure. Another difficulty is that buildings are occupied. Hence, the sensor network must be as minimally intrusive as possible. Nevertheless, necessary maintenance can still disturb inhabitants and data collection may be disturbed by inhabitants (moving or switching off sensors and gateways, for instance). Finally, retrofit actions are conducted during part of the instrumentation process, which results in several issues.
- *Targets of the sensor network.* Because the sensor network targets a large range of measurements, it is necessary to mix many different technologies of sensors, data acquisition, communication, and storage. These technologies are not always fully compatible and result in additional challenges for long-term project maintenance. Additionally, the sensor network relies on IoT objects. We observed that the current IoT market is more fitted for large-scale deployment strategies over approximately one year than for high-precision measurements and long-term monitoring.
- *Project management.* From the design brief to the installation and long-term maintenance, the sensor network requires optimized management, specifically regarding third parties, such as volunteering housings and contractors. Participants are essential to the project, since the results entirely depend on a sufficient amount and diversity of collected field data. Contractors also play a significant role. They usually provide “plug-and-play” management; sensors are provided, set up, installed, and maintained. However, most IoT contractors are energy managers who delegate installation and maintenance tasks to other contractors and have limited field knowledge. Hence, it is very time-consuming to solve any technical issue.

In addition to the published results [36], the installation of sensors in households has highlighted a few more insights into the challenges of building energy monitoring, specifically on the limitations of radio frequencies. LoRaWAN is the main communication protocol of our sensor network. It uses free radio frequencies and supposedly benefits from a “long range wide area” network. However, construction works have shown that the LoRaWAN network is easily disturbed, especially with scaffolding around the buildings that disrupted radio communication. The position of LoRa gateways has a significant impact on data communication, even with sensors and gateways within the same building. Several options could be considered. LoRa antennas located on a building roof are a simple and efficient strategy. However, it is difficult to link the antenna to the gateways, knowing that the sensors must be above or at the same level as the gateways. It could be possible to add more gateways, but there is an added cost and a lack of adapted locations onsite to secure the equipment. Switching to an operated LoRaWAN network would also be a solution, but with no guarantee that it could solve the problem. Moreover, some sensors would remain on the private LoRaWAN network because of acquisition time-steps and bandwidth usage, and the cost of data collection fees would be significant with an operated network.

### 4.2. Limitations and Potential Improvements

The objective of our sensor network was to provide an exhaustive characterization of energy consumption, IEQ, and inhabitants’ energy-driving behaviors in an occupied and retrofit group of three existing buildings. We achieved a significant challenge with the monitoring of twenty different parameters in housings, common areas, and energy systems by deploying a total of 170 sensors in the three renovated buildings (144 in apartments and 26 in common areas) and by collecting data for over three years. The implementation of the sensor network resulted in a significant amount of collected data that provided extensive knowledge on the operation of the studied buildings. Nevertheless, the sensor network still highlights several gaps that leave room for improvements.

#### 4.2.1. Measurements

Characterization of the occupants’ behavior is performed with presence detection and window/glazed door opening detection. Presence detection is achieved using a single sensor. One single presence detection sensor for each household limits the data acquisition strategy. The location of the sensor in the living room also constrains data analyses, despite the fact that volunteers declared spending most of their time in this specific room. Sensors perform presence detection but they cannot count the number of occupants in the room. To cope with these constraints, in terms of instrumentation, there would be complementary solutions, such as using additional sensors for presence detection in different rooms and monitoring of the entrance door opening. However, additional sensors would increase costs and potentially undermine inhabitant privacy protection. Housing occupancy can be deduced from other measurements, including CO${}_{2}$ concentration, electricity consumption of appliances, and window opening rates. This option can be considered but relates to data analyses rather than instrumentation solutions.

Comfort and indoor environment characterization can be improved as well. In the present study, it includes indoor air temperature, relative humidity, CO${}_{2}$ concentration, luminosity, and the indoor surface temperature of cold walls. Several aspects could be further explored with our sensor network, such as indoor air quality [37] (VOCs, air pollutants, PM2.5), the characterization of thermal comfort [38], or visual and noise disturbances [39] that can impact occupants’ behaviors and energy consumption.

Thermal energy consumption monitoring at the apartment scale was significantly constrained in our project. Because the main hot water pipes were not accessible, DHW and heating energy consumption were assessed with contact temperature measurements on DHW pipes and heaters. These measurements are useful to understand DHW and heating patterns. However, to translate to energy consumption, it requires other information—hot water flow, heater and pipe characteristics—that cannot, or can only partially, be collected in apartments. Therefore, several assumptions are necessary to assess the energy consumption. Another gap lies in the characterization of energy systems and building envelopes. Energy systems are considered with their energy consumption and through information extracted from documentation. The building envelope is known thanks to the retrofit project management portfolio. Depending on the energy system, there are many existing characterization techniques [40]. For building envelope characterization, several industrial and experimental methods have also been developed [41,42,43]. Nevertheless, most methods were too specific and costly for our research project.

Finally, spatial and temporal data granularity are essential aspects that could be improved. More sensors in more apartments with a smaller time granularity would offer more data opportunities. Nevertheless, choices were made to the best of our knowledge regarding the needs for data and energy analyses, the specificities of the experimentation site, and keeping in mind that the larger and the more detailed the sensor network is, the larger the challenge for day-to-day management, data processing, and long-term maintenance. With budget constraints and difficulties enrolling participating households, it was also decided to restrict the household sample and increase the number of sensors for each housing, rather than limiting the number of sensors and recruiting more volunteers. However, a limited eight-apartment sample calls into question the reliability of data analyses and replicability of the research work.

#### 4.2.2. Technological Choices

The choice of data to collect is critical to perform the expected energy analyses. Measurements are also impacted by available monitoring technologies and communication protocols. Technological choices are made based on the IoT market, the expertise of contractors, the needs of our research project, and the overall cost of the instrumentation solution.

The instrumentation solution aims to efficiently monitor the targeted measurements. However, sensor technologies can be discussed. For instance, pulse technology is a simple, affordable, and efficient means to collect energy consumption data. Meters with pulse output produce one pulse for each unit of consumed energy. The aggregated number of pulses for a given time-step is the information communicated by a pulse sensor connected to an energy meter. However, this technology does not provide the precision of meter readings and rounds the energy consumption for each time-step. Hence, electricity and thermal energy consumption monitoring can be improved. For the former, TIC technology (*Télé Information Client*, standing for remote client information) is an option. Instead of counting the number of pulses, it collects the same data as those sent to the electricity network administrator (energy index, current demand, power demand, active power). However, this technology was not available with the expected specifications through the contractors we selected and it would have been complicated to install and supervise these sensors on our own.

For thermal energy monitoring, because of the configuration of our building case study, ultrasonic thermal energy meters were selected. These meters can theoretically be installed and removed easily on any piping system. In practice, ultrasonic meters show strong limitations. Installation and calibration is complex. Indeed, ultrasonic probes must be carefully set up to ensure that the ultrasonic signal and flow calculation are reliable. It needs a steady and reliable flow with a clean piping system, and meters must be installed on pipe sections at least two meters long, without bending, size differences, or nearby pumping systems. Probes are very sensitive to dust, humidity, vibrations, and any handling that can significantly disturb the ultrasonic signal. Consequently, maintenance is advised every six months to ensure measurement quality. Finally, energy meters also embed a calculator to assess the energy consumption based on flow measurements, inlet–outlet temperature difference, and characteristics of the piping system. The latter are not always fully known. Therefore, integrated meters would have offered a more reliable measurement but could not be installed. The other option would have involved a Modbus [44] communication protocol to provide flow and temperature difference data along with the energy consumption.

Other sensor technologies can be targeted by potential improvement, including presence detection and window opening sensors. There are a variety of occupant counting technologies already used in industrial and commercial buildings [10]. These could be investigated with a focus on the preservation of privacy protection and a limited intrusivity of the monitoring solution. Window opening detection sensors provided relevant insights into occupant behavior. Nevertheless, a main issue is the impossibility of accurately assessing the amount of missing data because of the event-based data collection process. Furthermore, window opening detection translates to window opening and closing duration. There is no information on the opening width of the window. This information impacts on air flow between the inside and outside environments, which affects the IEQ, IAT, and heating energy demand.

Finally, communication protocols and equipment are an important part of the sensor network. LoRaWAN and GPRS both show pros and cons. The latter is reliable regarding punctual data loss and network dependency. However, it may have trouble accessing the network depending on the location of the sensors in the building. Moreover, because it transfers data upon collection, the life expectancy of batteries is rather short. LoRaWAN is the main communication protocol for our research project, divided between operated and private networks. The operated LoRa network is a “plug-and-play” solution. It is fully managed by operators and depends on a national LoRaWAN network. However, there are significant usage constraints regarding the amount of transmitted data. A private network is the obvious replacement solution. Nevertheless, it relies entirely on dedicated gateways. This is a precarious situation that results in data loss if any technical issues arise.

#### 4.2.3. Calibration and Long-Term Maintenance

Calibration and long-term maintenance are critical aspects to consider during the installation and supervision of a sensor network. They are essential to ensure the reliability of collected field data over the duration of the research project.

All sensors except for the thermal energy meters were calibrated by manufacturers before their installation. Thermal energy meters were calibrated upon installation.

To account for potential measurement drifts, several options are available. Occasional measurements onsite can be made for comparison with a calibrated sensor. If a difference is spotted between the collected data and punctual measurements, sensors can be removed and sent back to their manufacturer for calibration. In any case, onsite punctual measurements are challenging. They require measurements over several hours in apartments at different times over the research project, while one of the key aspects of the solution is to be as unintrusive as possible. If sensors are sent back to manufacturers, it results in a loss of data during calibration, unless removed sensors are momentarily replaced by other sensors that are specifically set up for this application. This solution was not implemented in our project because of the limited number of available sensors and the consecutive sensor management.

#### 4.2.4. Data Loss

A final aspect to improve in our sensor network is related to the missing data. Sensors were installed onsite and supervised to ensure that they measured and communicated the targeted data. Nevertheless, this does not mean that the collected data are flawless. There might be outliers (unexpected negative, zero, or abnormal data, or error messages) or missing data. These are related to different identified causes, such as:

- Installation conditions and environment: walls, scaffolding, metal elements, sensor location, or distance to the gateways;
- Unexpected sensors and gateway handling, from residents, construction teams, or visitors, willingly or accidentally;
- Electricity shortage affecting sensors and gateways with grid power supply;
- Equipment failure: worn out batteries, crashed sensors or gateways, either because of manufacturing defects, punctual bugs, overuse, or long-term usage defects;
- Network issues including difficulties for sensors or gateways to access GPRS or LoRaWAN networks;
- Server issues.

We observed that an efficient means to reduce the loss of data is to implement an automated data collection checking system. This was tested on part of our sensor network, with daily verification of the amount of collected data for a group of sensors, comparing the size of the daily data packages to the size of a normal data package, when there are no missing data. This solution could be further improved with a dedicated detailed process to check the amount of data received for each individual sensor, and to send warning e-mails in case of a suspected data loss.

## 5. Conclusions

We presented in this article the implementation and use of a wireless sensor network for energy monitoring for residential buildings’ energy performance assessment. All the steps are technically detailed from the design, deployment of sensors, communication protocols, and the data analysis to provide feedback for future deployments of wireless sensor networks in buildings. The proposed wireless sensor network was implemented in a field experiment covering three collective residential buildings located in the metropolitan area of Paris. The sensor network consists of large number of 179 sensors on a long-time monitoring period of a few years and a wide variety of parameters such as energy consumption, indoor environment quality, occupants’ behavior, and local meteorological conditions. The collected data are used to assess the performance of the buildings after refurbishment in terms of energy consumption and indoor environmental quality and to accurately characterize occupant behaviors and usages that are of particular interest to enrich building energy models. Observations from the collected data show energy consumption of the renovated buildings in agreement with expected energy savings calculated by an engineering office, many different occupancy patterns mainly related to the professional situation of the households, and seasonal variation in window opening rates. On the one hand, the CO${}_{2}$ data measured with the deployed wireless sensor network allowed us to check the correct functioning of the new ventilation system to ensure proper air renewal in apartments. On the other hand, monitoring was able to detect some deficiencies in energy management. Indeed, the data reveal the absence of time-of-day-dependent heating load control and higher-than-expected indoor temperatures because of a lack of occupant awareness of energy savings, thermal comfort, and the new technologies installed during the renovation such as thermostatic valves on the heaters. One of the objectives of the present work was to demonstrate the large benefits of monitoring for energy and indoor environmental quality purposes in occupied buildings. It is valuable for occupants, landlords, engineers, and researchers. Finally, we provided feedback on the performed sensor network regarding the experimental design, choice of measured quantities and data communication, as well as the technological choices, implementation, calibration, and maintenance of sensors. The lessons learned here will undoubtedly enable a better and wider use of such technologies for building energy performance enhancement.

## Figures and Tables

**Table 2:** Summary of apartment features in the three considered buildings.

Apt. Type	B1	B2	B3	nb.	Area (m${}^{2}$)
1 living room/bedroom (T1)	2	2	2	6	36
1 bedroom (T2)	4	5	7	15	50–53
2 bedrooms (T3)	4	10	14	28	63–58
3 bedrooms (T4)	3	4	6	13	74–79
TOTAL	13	21	29	63	/

**Table 3:** Description of the instrumented household sample. The dwelling types from T1 to T4 are those defined in Table 2. Floor 0 refers to the ground floor.

Building	Floor	Orientation	Type	Surface (m${}^{2}$)	Nb. of Occupants
B1	2	SE	T3	63	2
B1	3	NE	T2	50	1
B2	0	NW	T3	64	1
B2	1	SE	T2	53	1
B2	2	SE	T2	53	1
B2	5	SE	T2	50	1
B3	0	SE	T4	74	2
B3	2	SE	T3	70	1

**Table 4:** Description of occupants in the instrumented households. Occupation refers to the occupation of the head of the household.

Building	Floor	Nb. of Occupants	Occupation	Children	Pets
B1	2	2	Public servant	1	None
B1	3	1	Public servant	None	None
B2	0	1	Private sector employee	None	6
B2	1	1	Public servant	None	None
B2	2	1	Teacher	None	2
B2	5	1	Unemployed	None	2
B3	0	2	Retired	None	None
B3	2	1	Retired	None	1

**Table 5:** Details on the numbers and types of sensors in the different instrumented households (with one apartment per floor).

Building	B1		B2				B3		TOTAL
Sensors inside the Apartments									
Floor	2	3	0	1	2	5	0	2	
Clamp. Amp. meters	0	1	1	0	0	0	1	0	3
Elec. pulse sensor	1	1	1	1	1	1	1	1	8
plug	4	6	4	3	2	6	6	5	36
Gas pulse sensor	0	1	1	0	1	0	1	0	4
heater temp.	4	4	4	3	3	3	4	4	29
DHW pipe temp.	1	1	1	1	1	1	1	1	8
Indoor wall temp.	2	3	2	0	2	3	3	2	17
Temp., RH, lum., CO${}_{2}$, motion	1	1	1	1	1	1	1	1	8
Window opening	4	4	4	3	3	4	5	4	31
Total apartments	17	22	19	12	14	19	23	18	144
Sensors in common areas									
Clamp Amp. meters	0		0				1		1
Elec. pulse	2		3				2		7
Thermal energy	2		2				2		5
Temp., RH	3		3				3		9
Motion	1		1				1		3
Total common areas	6		8				8		26
TOTAL	**45**		**72**				**49**		**170**

**Table 6:** Summary of the accuracy and operating range of the weather station.

Sensors	Accuracy	Operating Range
Temperature	±0.3 °C (0 °C…+70 °C) 0.4 °C otherwise	−40…+105 °C
Humidity	±1.8% (0…85%, T = +15…+35 °C) ±2.5% (85…100%, T = +15…+35 °C) ±2+1.5% otherwise	0…100%
Rainfall	N.C.	N.C.
Solar irradiation	±10 W/m${}^{2}$	0…2000 W/m${}^{2}$
Wind speed	±2% (0…65 m/s), ±3% otherwise	0…80 m/s
Wind direction	±2°	0…359.9°
Atmospheric pressure	±0.5 hPa (800…1100 hPa, T = 25 °C)±1 hPa (300…1100 hPa, T = 0…50 °C)	300…1100 hPa
Radiant temperature	±0.1 °C	−200…+650 °C
Dew point temperature	N.C.	N.C.

**Table 7:** Summary of the accuracy and operating range of the deployed sensors in apartments and common areas.

Measurement Targets	Sensors	Accuracy	Operating Range
Electricity	Sensor with clamp ampere meters (Ewattch Tyness)	N.C.	N.C.
	Pulse sensor for electric smart meter (Fludia BelSenso FM410e)	N.A.	10 pulse/sec max
	Connected plug (NKEWatteco Smartplug)	>1% (P > 40 W) <1% (P < 40 W)	Voltage: 100…250 V Frequency: 50…60 Hz
Natural gas	Pulse sensor for gas meter (Adeunis Pulse ATEX)	N.A.	8 pulse/sec max
Thermal energy	Thermal energy meter for DHW and heating (Ultraflow U1000)	±1…3% (flow > 0.3 m/s)	Flow: 0.1…10 m/s Temperature: 0…+85 °C
	Contact temperature of heaters (SensingLab TEM-LAB-14NS)	±0.5 °C (−10…+85 °C) ±2 °C otherwise	Temperature: −45…+125 °C
	Contact temperature of DHW pipes (SensingLab TEM-LAB-14NS)	±0.5 °C (−10…+85 °C) ±2 °C otherwise	Temperature: −45…+125 °C
IEQ	Indoor temperature and humidity (SensingLab THY-LAB-41NS)	Temperature: ±0.3 °C Humidity: ±2%	Temperature: 0…+55 °C Humidity 0…80%
	Indoor temperature of cold walls (SensingLab TEM-LAB-14NS)	±0.5 °C (−10…+85 °C) ±2 °C otherwise	Temperature: −45…+125 °C
	Temperature	±0.2 °C (0…+60 °C)	−40…+120 °C
	Humidity	±0.2% (10…90%, T = 25 °C)	0…100%
	CO${}_{2}$	±50 ppm	0…2000 ppm
	Luminosity	±10 Lux	0…65,535 Lux
	(ELSYS ERSCO2)		
Occupants’ behavior	Presence detection	N.A	0…255 motions
	(ELSYS ERSCO2)		
	Presence detection (common areas) (SensingLab PIR-LAB-41NS)	N.A.	N.C.
	Window opening detection (SensingLab OPE-LAB-41NS)	N.C.	N.C.

## Data Availability

Data are not publicly available due to privacy and ethical restrictions. We do not have the occupants’ authorization to provide public access to the raw data.

## Abbreviations

The following abbreviations are used in this article:

AQC	Agence Qualité Contruction
APC	Agence Parisienne du Climat
BEMS	Building Energy Management System
CMV	Controlled Mechanical Ventilation
CNIL	Commission Nationale Informatique et Libertés
CSV	Comma Separated Values
CPCU	Compagnie Parisienne de Chauffage Urbain
CSTB	Centre Technique et Scientifique du Bâtiment
DHW	Domestic Hot Water
EDF	Electricité de France
EPD	Energy Performance Diagnosis
FTP	File Transfer Protocol
GPRS	General Packet Radio Service
GRDF	Gaz Reseau Distribution France
HVAC	Heating Ventilation Air Conditioning
HTTP	Hypertext Transfer Protocol
IAT	Indoor Air Temperature
IAQ	Indoor Air Quality
IEQ	Indoor Environment Quality
IoT	Internet of Things
LoRaWAN	Long Range Wide area network
NILM	Non Intrusive Load Monitoring
PIR	Passive InfraRed
QSE	Qualité Sanitaire et Energétique
SEREINE	Solution d’Evaluation de la peRformance Energétiuqe IntrinsèquE des bâtiments
TIC	Télé-Information Client
VOC	Volatile Organic Compound

## Appendix A. List of Sensors with Their References

**Table A1:** List of sensors with their references.

Sensor	Label	Documentation
Le compteur communicant|Enedis	Linky	https://www.enedis.fr/linky-compteur-communicant (accessed on 6 June 2023)
Compteur d’énergie thermique	EWATTCH	
LoRaWAN SMART PLUG	LoRaWAN SMART PLUG	http://www.nke-watteco.fr/wp-content/uploads/2018/06/LoRaWAN-Smart-Plug-Fiche-Technique-v1.1.pdf (accessed on 6 June 2023)
Capteur Tyness Energy	EWATTCH	https://www.ewattch.com/portfolio-835item/tyness-energy-lora/ (accessed on 10 April 2020)
PUL-LAB-13XS Outdoor ATEX LoRa	PUL-LAB-13XS Outdoor ATEX LoRa	SensingLabs
Compteur gaz communicant	Gazpar	https://www.grdf.fr/particuliers/fonctionnement-compteur-gaz-communicant-grdf (accessed on 6 June 2023)
Capteur Temperature-Hygrometrie LoRa	THY-LAB-61NS	SensingLab
ERS LoRaWAN room sensor for measuring indoor environment	ELSYS	https://www.elsys.se/en/ers/ (accessed on 6 June 2023)
Capteur PIR Indoor	PIR-LAB-21NS	SensingLab
Transmetteur contact magnetique outdoor	OPE-LAB-13NS	SensingLab
Transmetteur contact magnetique outdoor	OPE-LAB-13NS	SensingLab
Module temperature LoRa	TEM-LAB-14NS	SensingLabs
